# Diagnostic and Prognostic Value of miR-16, miR-146a, miR-192 and miR-221 in Exosomes of Hepatocellular Carcinoma and Liver Cirrhosis Patients

**DOI:** 10.3390/cancers13102484

**Published:** 2021-05-19

**Authors:** Thorben Fründt, Linda Krause, Elaine Hussey, Bettina Steinbach, Daniel Köhler, Johann von Felden, Kornelius Schulze, Ansgar W. Lohse, Henning Wege, Heidi Schwarzenbach

**Affiliations:** 1 Department of Medicine I, University Medical Center Hamburg-Eppendorf, 20246 Hamburg, Germany; e.hussey@uke.de (E.H.); j.von-felden@uke.de (J.v.F.); k.schulze@uke.de (K.S.); a.lohse@uke.de (A.W.L.); H.Wege@klinikum-esslingen.de (H.W.); 2 Institute of Medical Biometry and Epidemiology, 20246 Hamburg, Germany; l.krause@uke.de; 3 Department of Tumor Biology, University Medical Center Hamburg-Eppendorf, 20246 Hamburg, Germany; b.steinbach@uke.de (B.S.); hschwarzenbach@me.com (H.S.); 4 Department of Diagnostic and Interventional Radiology and Nuclear Medicine, University Medical Center Hamburg-Eppendorf, 20246 Hamburg, Germany; d.koehler@uke.de; 5 Cancer Center Esslingen, 73730 Esslingen am NEckar, Germany

**Keywords:** microRNAs, exosomes, hepatocellular carcinoma, liver cirrhosis, survival prediction

## Abstract

**Simple Summary:**

Exosomes, carrying small non-coding RNA (miRNA), are known to play a pivotal role in the process of tumor progression. In this prospective study, we identified miR-16, miR-146a, miR-192, and miR- 221 to be significantly deregulated in the plasma of patients with hepatocellular carcinoma (HCC) compared to patients with liver cirrhosis and healthy individuals. MiR-146a revealed diagnostic potential to differentiate HCC patients from liver cirrhosis patients with a sensitivity of 81% and a specificity of 58% in logistic regression model. Furthermore, miR- 192 independently correlated with overall survival in patients with HCC.

**Abstract:**

We aimed to identify a specific microRNA (miRNA) pattern to determine diagnostic and prognostic value in plasma exosomes of hepatocellular carcinoma (HCC) patients. A two-stage study was carried out: exosomal miRNAs were quantified in plasma of HCC patients and healthy individuals by PCR-based microarray cards containing 45 different miRNAs (training cohort). Then, four deregulated miRNAs (miR-16, miR-146a, miR-192, and miR-221) were quantified in the validation analysis using exosomes derived from 85 HCC patients, 50 liver cirrhosis patients, and 20 healthy individuals. Exosomal miR-146a (*p* = 0.0001), miR-192 (*p* = 0.002) and miR-221 (*p* = 0.032) were upregulated only in HCC patients. Repeated 10-fold cross validation showed that miR-146a differentiated HCC from liver cirrhosis patients with AUC of 0.80 ± 0.14 (sensitivity: 81 ± 13%, specificity: 58 ± 22%) in a logistic regression model. High miR-192 presence is associated with poor overall survival (OS) in all HCC patients (*p* = 0.027) and was predictor of OS in HCC patients in an uni- and multivariate Cox regression model. Moreover, decreased miR-16 levels correlated with OS in liver cirrhosis patients (*p* = 0.034). Our results emphasized that exosomes secreted into the plasma carry differentially expressed miRNAs of which in particular, miR-192, miR-146, and miR-16 are promising diagnostic and prognostic markers for both HCC and liver cirrhosis patients.

## 1. Introduction

Hepatocellular carcinoma (HCC) is the most common primary liver tumor and one of the leading cause of cancer related death worldwide [1]. The tumor primarily arises in patients with liver cirrhosis and it is estimated that approximately one third of patients with liver cirrhosis will develop liver cancer during their lifetime [2]. Prognosis of HCC is dismal because the diagnosis is often established in an advanced stage when tumor spread or vascular invasion hamper curative surgery or liver transplantation [3,4].

Of note, survival is impacted not only by the tumor burden, but also by the function of the underlying liver cirrhosis. Therefore, liver function is incorporated into the most relevant clinical classification systems (e.g., Okuda system, Barcelona Clinic Liver Cancer Classification (BCLC) and Cancer of Liver Italian Program (CLIP) classification). This illustrates the complexity of tumor therapy and the poor prognosis in patients with HCC and liver cirrhosis [5,6,7,8]. Furthermore, as therapeutic options for patients with advanced HCC have improved gradually over the past few years, it is crucial to identify prognostic markers predicting tumor progression and deterioration of the liver function in order to switch patients to more effective treatment lines [9,10,11,12].

MicroRNAs (miRNA) are small non-coding RNA molecules with a length of approximately 24 nucleotides. The main function of miRNAs is to inhibit the translation of their target mRNAs into protein. In this regard, they bind to complementary sequences in the 3′ untranslated-region (3′UTR) of their target mRNAs [13]. MiRNAs are released into blood circulation either passively by apoptosis and necrosis, or actively in exosomes from various cell types [14]. Exosomes are small membrane-embedded vesicles of about 100 nm, mediating cell-to-cell communication by transferring their oncogenic cargo. Exosomal miRNAs can modulate the genotype and phenotype of the recipient cell due to their specific function, that is, i.e., by altering cellular signal pathways and gene expression and inducing tumor progression and metastasis [14]. In this regard, levels of secreted exosomes have been associated with tumor invasiveness both in vitro and in vivo and are, therefore, of great interest as promising biomarkers [15].

In the present study, we investigated the diagnostic and prognostic relevance of miR-16, miR-146a, miR-192, and miR-221 in HCC and liver cirrhosis using two different analytical methods. We also demonstrate their differential expression in plasma exosomes in a cohort of patients with liver cirrhosis and HCC.

## 2. Materials and Methods

### 2.1. Study Populations

This study included a total of 84 HCC patients and 50 liver cirrhosis patients, who were treated at the I. Department of Medicine of the University Medical Center Hamburg-Eppendorf between November 2017 and July 2019. Liver cirrhosis was diagnosed either histologically or by non-invasive assessment of liver stiffness (e.g., Fibroscan^®^ (Ecosense, Paris, France); a mean value from 10 valid measurements > 16 kPa was considered significant) and imaging criteria (e.g., nodular margin of the liver, hypertroph of lobus caudatus, or lobar atrophy, signs of portal hypertension in the absence of portal vein thrombosis, ascites). The presence of ascites was diagnosed on admission or presentation to outpatient department either by ultrasound or by radiology imaging (e.g., CT, MRI) when fluid in abdominal cavity was present without evidence of portal vein thrombosis. Hepatic encephalopathy was diagnosed in patients presenting with cognitive impairment in the absence of underlying neurologic or psychiatric disease by psychometric test according to recent guidelines [16]. Hepatorenal syndrome was diagnosed according to the criteria of the International Club of Ascites as recommended in recent guidelines [17,18].

Diagnosis of HCC was histologically confirmed or based on defined imaging criteria according to recent guidelines [19]. Tumor staging was performed according to the BCLC classification system and the study only included patients classified as having early (BCLC A), intermediated (BCLC B) or advanced stage (BCLC C). Treatment modalities comprised trans-arterial chemoembolization (TACE] for stage A and B HCC patients, and systemic therapy using sorafenib, lenvatinib or cabozantinib for stage C patients. Written informed consent was obtained from all participants prior to any study procedure. Blood specimens were obtained by venous puncture and were immediately centrifuged at 3600 rpm at room temperature for 10 min. Plasma samples were stored at −80 °C prior to analysis. Blood collection and experiments were performed in compliance with the Helsinki Declaration and were approved by the local Ethics Committee (Ethik-Kommission der Ärztekammer Hamburg, Hamburg, PV-3578). For quantification of exosomal miRNAs using array cards containing 47 miRNAs, we analyzed plasma samples from 24 HCC patients, 37 liver cirrhosis patients, and 20 healthy individuals (training cohort, clinical parameters are listed in Table 1). In a second step, a validation cohort was established for single cell analysis. This cohort was made up 84 HCC patients, 50 liver cirrhosis patients and 20 healthy controls, resulting in a total amount of 154 patients (validation cohort, clinical parameters are depicted in Table 2). Follow-up was completed on 18 November 2019. Electronic patient files were reviewed to assess the clinical course. The period of follow-up was determined based on the date when blood specimens were obtained upon patient’s death or the previous follow-up.

### 2.2. Verification of Hemolysis in Plasma Samples

To avoid quantifying exosomal miRNAs in hemolytic plasma samples that may influence our results, we performed hemoglobin measurements by spectral analysis [20]. In 7 mL of whole blood, red blood cells were lysed by erythrocyte lysis buffer (containing 0.3 M sucrose, 10 mM Tris pH 7.5, 5 mM MgCl2, and 1% Triton ×100). A dilution series (undiluted; diluted 1:1, 1:3, 1:4, 1:6, 1:8, 1:10, 1:12, 1:14, 1:18, 1:20 with plasma] of lysed red blood cells in plasma was prepared that served as a standard curve for the measurement of hemolysis in all plasma samples. Then, 50 µL of each plasma sample (standard and plasma of interest) were measured in duplicates on a Microplate reader (Tecan, Männerdorf, Switzerland). Absorbance peaks at 414, 541, and 576 nm were indicative for free hemoglobin, with the highest peak at 414 nm. The higher the absorption in samples, the higher is the degree of hemolysis. The average values and standard deviations were calculated from the duplicates (Supplementary Figures).

### 2.3. Exosome Extraction

Exosomes were isolated from plasma samples with the ExoQuick kit (BioCat, Heidelberg, Germany). Briefly, 500 µL plasma was removed from cells and debris by two centrifugation steps at 3000× *g* for 15 min., and incubated with 120 µL ExoQuick exosome precipitation solution at 4 °C, for 30 min. Following centrifugation at 1500× *g* for 30 min. and at 1500× *g* for 5 min., the pellet contained the exosomes.

### 2.4. Western Blot

The protein concentrations were measured with the DC Protein Assay Kit (BioRad, Munich, Germany) at a wavelength of 650 nm on a spectrophotometric plate reader (Tecan). A standard curve of 0, 0.15625, 0.3125, 0.625, 1.25, 2.5, 5, and 10 mg/mL bovine serum albumin (BSA; Sigma–Aldrich Chemie, Munich, Germany) was applied by the double-dilution method. Then, 2.5 μL of exosomes and BSA standard samples, all resuspended in Phosphate-Buffered Saline (PBS) buffer (Life Technologies, Paisley, UK) were added to 96-well plates according to the manufacturer’s instructions. The protein concentrations were calculated according to a linear equation of y = mx + n by applying the linear regression method. For Western blot, 30 μg of exosomes resuspended in PBS buffer (Life Technologies) were electrophoretically separated and blotted onto a polyvinylidene difluoride (PVDF) membrane (Millipore, Billerica, MA, USA) which was subsequently incubated with an antibody specific for the exosomal marker CD63 (Abgent, San Diego, CA, USA) overnight. Detection of the proteins was carried out using a peroxidase-conjugated secondary antibody (Dako, Glostrup, Denmark) and a chemiluminescence ECL detection solution (Sigma–Aldrich, St. Louis, MO, USA).

### 2.5. Extraction of miRNAs from Exosomes

MiRNAs were extracted from exosomes resuspended in 150 µL lysis buffer (Thermo Fisher Scientific, Vilnius, Lithuania) and 50 µL PBS (Life Technologies] by using the TaqMan miRNA ABC Purification Buffer Kit (Thermo Fisher Scientific). According to the manufacturer’s instructions, the exosomal miRNAs were bound to 80 µL anti-miR beads using the TaqMan miRNA ABC Purification Bead kit Human panel A (Thermo Fisher Scientific). To avoid technical variability, 2 µL of 1 nM synthetic non-human cel-miR-39 were added as an exogenous spike in control.

### 2.6. Conversion of Exosomal miRNAs into cDNA

Reverse transcription was carried out using modified protocols of TaqMan MicroRNA Reverse Transcription kit (Thermo Fisher Scientific). For PCR-based TaqMan miRNA array, the reaction contained 6.0 µL Custom RT Primer Pool, 0.3 µL dNTPs with 100 mM dTTP, 3.0 µL (50 U/μL) MultiScribe Reverse Transcriptase, 1.5 µL 10xRT Buffer, 0.19 µL (20 U/μL) RNase Inhibitor and 4 µL exosomal miRNAs. For single TaqMan PCR analyses, the reaction contained 4.0 µL RT Primer Pool (RT primer of miR-484, cel-miR-39, miR-16, miR-30b, and miR-93 mixture diluted in Tris-EDTA (TE) 1:100), 0.2 µL dNTPs with 100 mM dTTP, 2.0 µL (50 U/μL) MultiScribe Reverse Transcriptase, 1 µL 10xRT Buffer, 0.127 µL (20 U/μL) RNase Inhibitor, and 2 µL exosomal miRNAs. The reactions were carried out at 16 °C for 30 min, 42 °C for 30 min and 85 °C for 5 min on an MJ Research PTC-200 Peltier Thermal Cycler (Global Medical Instrumentation, Ramsey, MN, USA).

### 2.7. Preamplification of cDNA

To increase input of cDNA, a preamplification step of cDNA was included. For PCR-based TaqMan miRNA array analyses, 25 µL preamplification reaction mix contained 12.5 µL TaqMan PreAmp Master Mix, 3.75 µL Custom PreAmp Primer Pool (Thermo Fisher Scientific) and 5 µL cDNA. For single TaqMan PCR analyses of miR-16, miR-30b, and miR-93, cDNA of the reference miR-484 and miR-39 was also preamplified. Here, 1 μL cDNA were preamplified in 5 μL Taqman PreAmp Master Mix (Thermo Fisher Scientific) and 1.5 μL PreAmp primer pool (TaqMan miRNA primers of miR-484, cel-miR-39, miR-16, miR-30b, and miR-93 mixture diluted in TE 1:100). The reactions were run on an MJ Research PTC-200 Peltier Thermal Cycler (Global Medical Instrumentation): 1 cycle at 95 °C for 10 min, 55 °C for 2 min, 72 °C for 2 min; 16 cycles at 95 °C for 15 s, 60 °C for 4 min; and a final cycle at 99.9 °C for 10 min. A negative control without any templates was also included from the starting point of reverse transcription.

### 2.8. PCR-Based TaqMan miRNA Arrays

Custom real-time PCR-based TaqMan miRNA array cards (Thermo Fisher Scientific) were used for miRNA profiling. These array cards contain assays to detect 45 human miRNAs of interest, 1 endogenous reference miRNA (miR-484), and 1 exogenous reference miRNA (cel-miR-39) for data normalization and 1 assay with an N/A-4343438-Blank (negative control). For the array cards, we selected the 45 miRNAs of interest because they have previously been described to be clinically relevant in cancer, with an exclusive consideration for HCC. These miRNAs of interest were then mounted on the array cards by the company Thermo Fisher Scientific and are as follows: RNU6, miR-9, miR-10b, miR-15b, miR-16, miR-18a, miR-19a, miR-20a, miR-21, miR-23a, miR-23b, miR-24, miR-27b, miR-31, miR-34c-3p, miR-92a, miR-96, miR-101, miR-106b, miR-107, miR-122, miR-130b, miR-135a, miR-143, miR-146a-5p, miR-155, miR-181a, miR-182, miR-183, miR-192, miR-210, miR-221, miR-222, miR-223, miR-224, miR-330-5p, miR-367, miR-374, miR-425-5p, miR-454, miR-484, miR-494, miR-519a, miR-522, miR-548a-5p, miR-888-5p.

For miRNA array analyses, we modified the protocol of Thermo Fisher Scientific as follows: The 112.5 µL PCR reaction contained 56.25 µL TaqMan Universal Master Mix II and 2 µL PreAmp product. PCR array cards were run on a 7900 HT Fast Real-Time PCR System (Applied Biosystems): 1 cycle at 95 °C for 10 min, 40 cycles at 95 °C for 15 s, and 60 °C for 1 min.

### 2.9. Single TaqMan PCR Analyses

For quantitative real-time PCR, the TaqMan miRNA assays (Thermo Fisher Scientific) for miR-484 and cel-miR-39 (reference miRNAs), and miR-16, miR-146a, miR-192, and miR-221 were used. In a 10 μL-reaction, 0.5 μL preamplified cDNA were mixed with 5 μL TaqMan Universal PCR Master Mix and 0.5 μL TaqMan MicroRNA Assay Quantitative real-time PCR reaction was performed at 95 °C for 10 min and in 40 cycles at 95 °C for 15 s and 60 °C for 60 s, on a C1000 Touch real-time PCR device (Bio-Rad, Hercules, CA, USA).

### 2.10. Data Normalization and Statistical Analyses

Data analyses were performed using the Thermo Fisher Scientific Analysis Software, Relative Quantification Analysis Module, version 3.1 (www.aps.thermofisher.com, accessed on 9 May 2019), and SPSS software package, version 22.0 (SPSS Inc., Chicago, IL, USA).

As there is no consensus on a reference miRNA for data normalization, we chose exosomal miR-484 and cel-miR-39 as an endogenous and exogenous reference geneto normalize our miRNA data. MiR-484 showed the smallest variation between healthy individuals, HCC patients, and liver cirrhosis patients. The inter-individual variability of the efficiency of our procedures was controlled by spiking of cel-miR-39-3p. The obtained data of the miRNA expression levels were calculated by the ΔCt method as follows: ΔCt = mean value Ct (reference cel-miR-39 + miR-484) − mean value Ct (miRNA of interest). The Thermo Fisher Scientific Analysis Software was used for performing hierarchical clustering (heat map) and volcano plots: To detect potential clusters in rows (miRNAs) and columns (plasma samples) of the normalized expression matrix, hierarchical clustering was performed derived from analyses of the array cards. The ΔCt values of miRNAs vs. the mean of references miR-484 and cel-miR-39 among all 81 samples that were analyzed using the micro array cards were median-centered and clustered by unsupervised hierarchical clustering based on average linkage and Pearson’s correlation as distance metric. The similarity matrix contains all pairwise similarities of the exosome samples from plasma of HCC patients, liver cirrhosis patients and healthy controls. Subsequently, the relative expression data were 2^(ΔCt) transformed in order to obtain normal distribution data. The confidence of 2^(ΔCt) data were verified by amplification curves and Ct confidence (0–1, whereby 1 refers to the highest confidence). Our data showed a Ct confidence of 0.95. Statistical difference of exosomal miRNA expressions between healthy controls, HCC patients, liver cirrhosis patients, and healthy individuals were calculated using two-tailed student t-test, corrected according to the Benjamini and Hochberg method and depicted as a volcano plot.

For nonparametric comparisons of two independent variables, miRNA differences in group levels were compared by the Mann–Whitney-U test. Correlations of miRNA levels with the clinical parameters were calculated using ANOVA with Tukey’s HSD test for all pairwise comparisons that correct the experiment related error rate. Two-sample comparisons were performed using student’s t-test for equal or unequal variance where appropriate. Univariate and multivariate analyses were performed for prognostic factors of OS using the Cox regression model. Kaplan-Meier plots were drawn to estimate OS, and the log-rank test was applied for statistical analyses. Missing data were handled by pairwise deletion. *p*-values below 0.05 were considered statistically significant. All p-values are two-sided. Discriminating HCC from liver cirrhosis patients was modeled from logarithmized miR146a concentration using logistic regression and repeated (50 repeats) 10-fold cross validation using the R package caret version 6.0-86 (https://CRAN.R-project.org/package=caret, accessed on 14 May 2020). Model performance was evaluated using receiver operator characteristics (ROC) curve and summarized with mean and standard deviation of area under the ROC curve (AUC), as well as sensitivity and specificity for the results of the repeated cross-validation runs.

## 3. Results

### 3.1. Verification of Plasma Samples and Exosomes

**Figure 1:**
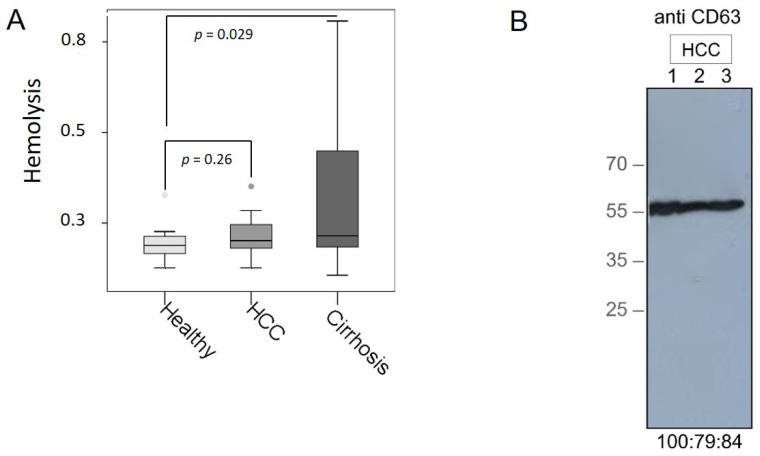
Verification of plasma samples and exosomes. Hemolysis was assessed by spectrophotometry at wavelengths from 350 to 650 nm. The degree of hemolysis was determined based on the optical density (OD) at 414 nm (absorbance peak of free hemoglobin, called Soret band), with additional peaks at 541 and 576 nm. Samples were classified as being hemolyzed if the OD at 414 exceeded 0.3. The box blot shows the levels of hemolysis in plasma samples from HCC and liver cirrhosis patients compared with those from healthy individuals with the *p*-values (**A**). Exosomes were precipitated from 3 plasma samples of HCC patients by the agglutinating agent ExoQuick and analyzed by a Western blot using an antibody specific for the exosomal marker CD63. The percentages under the blot show intensities of the areas of three bands (**B**).

To avoid analyzing hemolytic plasma samples, we carried out a hemolysis assay. As expected, plasma samples from HCC patients and healthy individuals were not hemolytic. However, the measurements of hemolysis in the plasma samples from liver cirrhosis patients were affected by hyperbilirubenimia in plasma samples (*p* = 0.029, Figure 1A). Therefore, hemolysis could not be determined in these samples, but these results show an interesting aspect of the extent of bilirubin secretion into the plasma. Extraction of exosomes from three plasma samples was verified by Western Blot using an antibody specific for the exosomal marker CD63. As shown by the 45-kDa band on the blot, the CD63-specific antibody recognized non-lysed exosomes in our samples (Figure 1B). Additionally, extraction of exosomes has already been confirmed by our previous study visualizing exosomes by confocal microscopy [21].

### 3.2. MiRNA Profiling in Exosomes Using Microarray Cards

**Figure 2:**
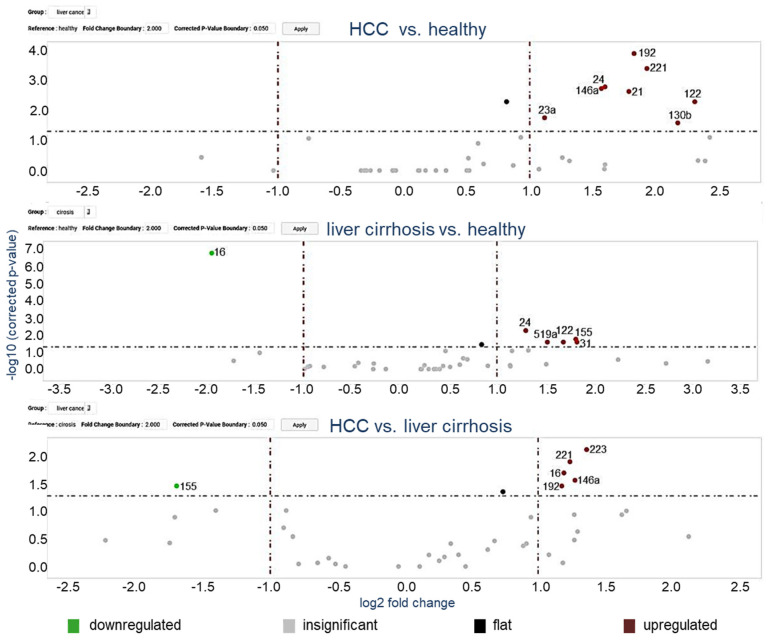
Volcano plot of 45 exosomal miRNAs. The volcano plots of *p*-values vs. fold changes compare the levels of exosomal miRNAs in 24 HCC patients and 37 liver cirrhosis patients with 20 healthy individuals, as well as 24 HCC patients with 37 liver cirrhosis patients. The grey dashed line refers to the threshold value corresponding to a corrected *p*-value of *p* = 0.05. Significantly downregulated exosomal miRNAs are shown as green dots, significantly upregulated exosomal miRNAs as red dots. Grey dots represent non-significant changes. *p*-values are calculated by the student *t*-test and corrected according to the Benjamini and Hochberg method.

In a two-step analysis, we first quantified miRNAs in exosomes derived from the plasma of 24 (14 early and 10 advanced) HCC patients, 37 liver cirrhosis patients and 20 healthy individuals (training cohort). MiRNAs were selected as listed in Materials and Methods for the assembly of the 48-microarray cards based on our previous studies [22,23,24,25] and research in PubMed based on their significant deregulation in HCC, and with preference to upregulated oncogenic miRNAs. Expression analysis identified clusters of up- and downregulated miRNAs (heat map, Figure S1. The relative upregulatedand downregulated miRNAs are indicated by red and green, respectively). Using the ΔCt method to determine relative expression, we demonstrated that eight and five miRNAs were significantly enriched in exosomes from plasma of HCC and liver cirrhosis patients, respectively, as compared with those of healthy individuals (Figure 2). The levels of six exosomal miRNAs could discriminate between HCC and cirrhosis patients (Figure 2). For a better overview, a Supplemental Figure (Figure S2) depicts the results as box plots, and additionally, the difference between early and advanced HCC patient subgroups is shown. Significantly deregulated miRNAs (*p*-values below 5%) calculated from the array cards are depicted in Tables S1 and S2. In particular, miR-16 was decreased in exosomes from cirrhosis patients (*p* = 0.0001) and miR-16 levels were differentially expressed between patients with and without liver cirrhosis (*p* = 0.019). The levels of exosomal miR-146a (*p* = 0.002), miR-192 (*p* = 0.0001) and miR-221 (*p* = 0.0004) were significantly increased in HCC patients, but not deregulated in cirrhosis patients, and, therefore, could discriminate HCC from cirrhosis patients, suggesting their HCC-specific enrichments in exosomes. Of note, the levels of exosomal miR-21 (*p* = 0.002) were only increased in HCC patients and could not discriminate between patients with and without HCC while the levels of miR-24 (*p* = 0.002, *p* = 0.005, respectively) and miR-122 (*p* = 0.005, *p* = 0.026, respectively) were enriched in exosomes from both HCC and cirrhosis patient cohorts compared to healthy patients (Table S1, Table S2, Figure S2). Based on the data obtained with array cards, we selected miR-16, miR-146a, miR-192, and miR-221 for further analyses using single TagMan real-time PCR assays.

### 3.3. Exosomal miR-16, miR-146a, miR-192, and miR-221 Profiling Using Single TaqMan Real-Time PCR Assays

**Figure 3:**
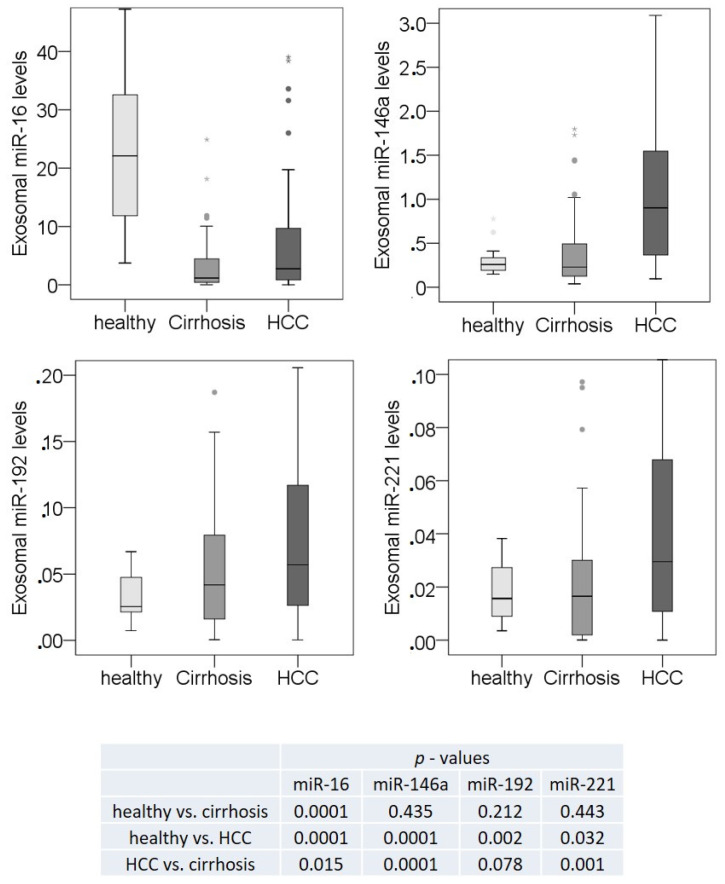
Significant deregulation of exosomal miR-16, miR-146a, miR-192 and miR-221. The box plots compare the exosomal levels of miR-16, miR-146a, miR-192, and miR-221 in the plasma of 84 HCC patients, 50 liver cirrhosis patients, and 20 healthy individuals, as derived from the data of single TaqMan real-time PCR assays. The significant *p*-values of the statistical evaluations are summarized in the table below the box blots. * *p* < 0.05; ** *p* < 0.001. ·, ··, extreme values.

We analyzed the expression of miR-16, miR-146a, miR-192, and miR-22 using single TaqMan real-time PCR in the training cohort and in plasma samples from an additional 60 HCC patients and 13 patients with liver cirrhosis, leading to a total number of 84 HCC patients, 50 liver cirrhosis patients, and 20 healthy individuals (validation cohort). As shown in Figure 3 and Table S2, right panels, the significantly lower levels of exosomal miR-16 detected in the plasma of liver cirrhosis patients compared to healthy individuals using the array cards were confirmed by the single target analyses with RT-PCR (*p* = 0.0001). The difference in the levels of this miRNA between HCC and cirrhosis patients were also similar using both techniques (*p* = 0.015, 0.019, Table S2). In contrast to array cards, the single TaqMan PCR assays with a larger HCC cohort showed significantly lower levels of exosomal miR-16 in HCC than in healthy individuals (*p* = 0.0001). The single TaqMan PCR assays also confirmed that the levels of exosomal miR-146a, miR-192, and miR-221 were upregulated in HCC patients compared with healthy individuals (*p* = 0.0001, *p* = 0.002, *p* = 0.032, respectively) and with cirrhosis patients (*p* = 0.0001, *p* = 0.078, *p* = 0.001, respectively), whereas they were not deregulated in cirrhosis patients (Table S2 and Figure 3).

### 3.4. Correlations of Exosomal miR-16, miR-146a, miR-192, and miR-221 with the Clinicopathological Parameters

To determine diagnostic value of miR-16, miR-146a, miR-192, and miR-221 logistic regression models were calculated using repeated cross-validation and evaluated using receiver operating characteristics (ROC) curves. From the four miRNAs, miR-146a showed an area under the ROC curve (AUC) of 0.80 ± 0.1476 with a sensitivity of 81 ± 13% 61% and a specificity of 78% 58 ± 22%. In terms of diagnosis it was alsosuperior to miR-16 (ROC AUC 0.63 +/− 0.16; sensitivity 88% +/− 12%, specificity 7.6% +/− 11%), miR-221 (ROC AUC 0.69 +/− 0.14; sensitivity 90% +/− 11%, specificity is 27% +/− 19%), and miR-192 (ROC AUC is 0.59 +/− 0.16; sensitivity 93% +/− 9.0%, specificity is 10% +/− 13%). A combination of the log values of all 4 miRNAs demonstrated an identical sensitivity (ROC AUC 0.78 +/− 0.13, sensitivity 81% +/− 14%, specificity 56% +/− 22%), but was less specific when compared to miR-146a. Compared with the biomarker AFP, no significant correlation was found in pairwise spearman correlation including AFP and all four selected miRNA (Table S3).

In patients with liver cirrhosis, expression miR-146a, miR-192, and miR-221 was significantly lower in patients with compensated cirrhosis compared to decompensated liver cirrhosis (e.g., presence of ascites, hepatorenal syndrome (HRS) and hepatic encephalopathy (HE)): miR-146a and miR-192 were significantly decreased in patients with ascites (*p* = 0.01 and 0.04, respectively). Furthermore, in patients with HRS, miR-16 (*p* = 0.02) and miR-146 (*p* = 0.002) were significantly decreased while only miR-146a (*p* = 0.03) was decreased in patients with HE. In HCC patients, expression of miR-16 significantly differs between tumor stage BCLC A, B and C/D (*p* = 0.004) using Kruska-Wallis rank sum test. No significant difference was found in the expression of miR-16 (*p* = 0.611), miR-146a (*p* = 0.206), miR- 192 (*p* = 0.127), and miR-221 (*p* = 0.596) of patients with alcoholic vs. non-alcoholic liver cirrhosis.

### 3.5. Prognostic Relevance of miR-192, miR-146a for HCC and miR-16 for Liver Cirrhosis

**Figure 4:**
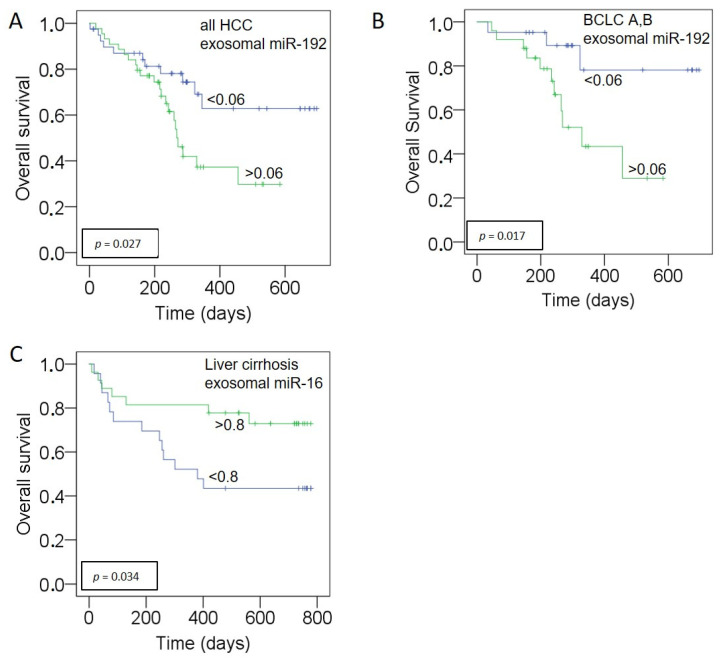
Association of exosomal miR-192 and miR-16 with poor outcome. Kaplan–Meier analyses show significant correlations of increasing levels of exosomal miR-192 in all HCC patients (**A**) and in patients of BCLC stage A and B (**B**). Decreased levels of exosomal miR-16 in liver cirrhosis patients significantly correlate with poor OS (**C**). As determined by the log-rank test, the significant *p* values of the statistical evaluations are indicated at the curves.

**Figure 5:**
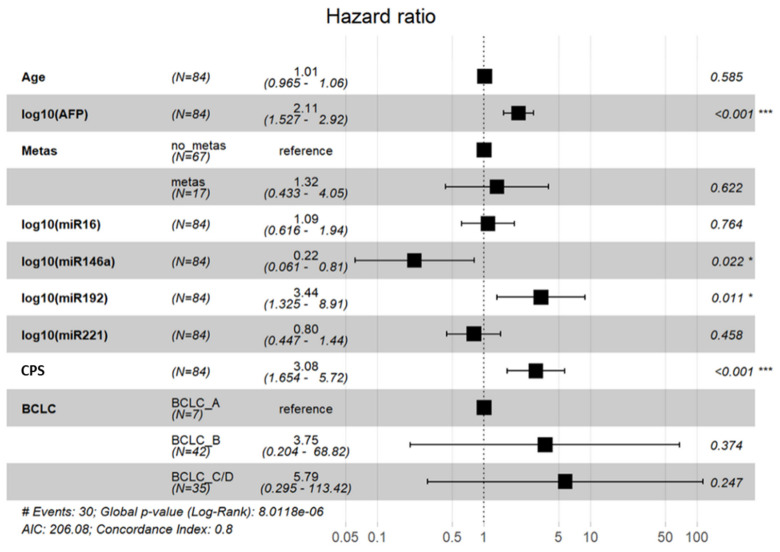
Cox regression model. Abbreviations: AFP, alpha-fetoprotein; Metas, presence of distant metastases; Child: child pugh score; BCLC: Barcelona Clinic for Liver Cancer. * *p* < 0.05; *** *p* < 0.001.

Kaplan–Meier and log-rank models were carried out to assess the prognostic potential of our miRNA panel in HCC and liver cirrhosis patients. The median follow-up time was 246 days (range: 2 to 697 days) for HCC patients and 543 days (range: 9 to 778 days) for liver cirrhosis patients. Median values of exosomal miRNAs were used for grouping the miRNAs according to their low and high presence in exosomes. As shown in Figure 4, high levels of exosomal miR-192 correlated with poor OS in all HCC patients (*p* = 0.027, log-rank test, A) and the HCC subgroup of BCLC stage A and stage B patients (*p* = 0.017, A). No correlation could be found in the subgroup of BCLC C. Univariate analysis with the Cox proportional hazards showed that exosomal miR-192 was a prognostic factor (*p* = 0.031, HR: 2.241, 95% CI: 1.079–4.656) for overall survival. In multivariate Cox regression analysis, the levels of miR-192 (HR: 3.44, 95% CI: 1.32–8.91; *p* = 0.01), miR-146a (HR: 0.22; 95% CI: 0.061–0.81, *p* = 0.02), Child Pugh Score (HR: 3.08; 95% CI: 1.65–5.72, *p* < 0.001), and AFP (HR: 2.1; 95% CI: 1.52–2.92, *p* < 0.001) were independent predictors of OS in HCC patients (Figure 5). As depicted in Figure 4, in cirrhosis patients, the low levels of exosomal miR-16 correlated with poor OS (*p* = 0.034) while multivariate regression analysis including patients age, miR-16, miR-146a, miR-192, miR-221, and MELD-Score only revealed MELD score as independently associated with OS (HR 1.1; 95% CI: 1.03–1.2, *p* = 0.007).

## 4. Discussion

Emerging evidence indicates that exosome-mediated transfer of miRNAs may contribute to the development and progression of HCC [26]. Our study served to gain a better understanding on this. To this end, we first identified a panel of significantly deregulated miRNAs in both, liver cirrhosis and HCC patients. For further single miRNA analyses, we chose miR-16 because of its cirrhosis-specific lower packing into exosomes and miR-146a, miR-192, and miR-221 because of their HCC-specific expression. Our single TaqMan PCR assays confirmed our array data, demonstrating that all four exosomal miRNAs were deregulated in HCC patients.

The most noteworthy finding of our study was that the plasma levels of exosomal miR-192 had diagnostic and prognostic value in our HCC patient cohort and that exosomal occurrence of miR-192 was associated with decreased overall survival (OS). In line with these finding, Xue et al. [27] showed elevated levels of exosomal miR-192 in serum of HCC patients. They, however, did not determine any association with OS. There may be several reasons for the discrepancy between Xue et al. and our result. Xue et al. used serum instead of plasma. Anticoagulants required for the preparation of plasma, such as heparin, acid citrate dextrose (ACD), or EDTA agents, and preanalytical variables may have brought about a difference in the data [28]. Nowadays, the use of plasma is preferred. Another reason might be the different spectrum of underlying liver disease in the cohort of Xue et al. compared to our cohort: in the Asian cohort, the majority of patients had chronic viral hepatitis and liver cirrhosis was present only in 37% of the patients, while the main etiology of liver cirrhosis in our cohort was alcoholic liver disease. Furthermore, Zhu et al. [29] showed that high serum levels of exosome- and cell-free circulating miR-192 were associated with poor OS. Thus, the enrichment of miR-192 in exosomes can add prognostic value, especially for patients in an early or intermediated tumor stage because these patients were treated with surgical resection, microwave ablation (MWA) or TACE. Suheiro et al. recently demonstrated that alterations in the expression of exosomal miR-122 is associated with survival in HCC patients treated with TACE, underlining the ability of miRNAs to serve as biomarkers for therapy monitoring [30]. Detecting deregulated miR-192 in plasma of HCC patients may, therefore, be useful to characterize patients with poor prognosis who could be considered for adjuvant therapy or who may benefit from an early transition to systemic therapy.

MiR-16 is known to be downregulated in HCC cells and its overexpression inhibits proliferation, invasion, and metastasis of HCC cells [31], suggesting that miR-16 acts as a tumor suppressor. MiR-16 is able to suppress HCC cell progression by targeting the immunogenic protein faint expression in normal tissues, aberrant overexpression in tumors (FEAT) and inhibiting epithelial-mesenchymal transition (EMT) and the nuclear factor-κB (NF-κB) [32]. Therefore, the use of miR-16 as internal reference should be circumvented for miRNA data normalization [33,34]. In our study, we found lower levels of miR-16 in exosomes of HCC patients compared to healthy individuals. Expression levels of miR-16 was also associated with BCLC staging and tumor metastasis. As far as we know, miR-16 has not yet been quantified in exosomes from HCC patients. However, there are some studies on circulating miR-16 in serum of HCC patients. To this regard, Ge et al. [35] detected that miR-16 expression was down-regulated in HCC patients with a tumor more than 5 cm in diameter and correlated with quantitative clinical features, such as platelet counts and serum bilirubin. Qu et al. [36] also observed decreased serum levels of miR-16 in HCC and their association with tumor size. Taken together, our findings indicate the role of miR-16 as a surrogate marker for tumor progression and dissemination. In addition, we found that exosomal miR-16 is both a diagnostic and prognostic factor in liver cirrhosis patients. In our study, we show for the first time in liver cirrhosis patients an under-presentation of miR-16 in exosomes in non-cancerous liver disease. A deregulation of miR-16 was also detected in a liver fibrosis animal model and patients with severe fibrosis. As demonstrated by Kim et al., downregulation of miR-16 promoted progression of liver fibrosis via activated hepatic stellate cells [37]. Advanced fibrosis and alterations of the hepatic architecture led to an increase in portal vascular resistance and finally, portal hypertension [30]. As portal hypertensions often resulted in development of esophageal varices and ascites, representing severe complications of the liver cirrhosis, this might be explanation for the prognostic impact of miR-16.

The role of miR-146a in patients with HCC is controversial: recent studies have reported that miR-146 acts as both an oncogene and a tumor suppressor gene, participating in several pathogenic pathways associated with hepatocarcinogenesis [38]. As an oncogene, miR-146a seems to play a key role in regulating the angiogenic activity of endothelial cells in HCC through its participation in the platelet-derived growth factor receptor α-BRCA1 pathway [39]. In addition, in HCC patients, upregulated miR-146a expression contributed to natural killer cell dysfunction by targeting signal transducer and activator of transcription 1 (STAT1) [40]. Interestingly, Yin et al. showed that HCC-derived exosomes could remodel macrophages by activating NF-κB signaling and inducing pro-inflammatory factors. These exosomes were enriched with miR-146a and promoted M2-polarization of tumor-associated macrophages [41]. In our study, miR-146a is enriched in exosomes of HCC patients, but not in liver cirrhosis patients. Multivariate analysis revealed that exosomal enrichment of miR-146a is statistical significantly associated with reduced hazard ratio for death, strongly suggestion that miR-146a primarily acts as a tumor suppressor. Furthermore, we demonstrated that the levels of miR-146a can distinguish between cirrhotic patients with and without HCC with a ROC/AUC of 0.80, comparable to the established tumor marker AFP (ROC/AUC: 0.83) [42]. As tumor markers are urgently needed for HCC, the promising diagnostic function of miR-146a should be further evaluated in a larger cohort of patients.

MiR-221 is an oncogenic miRNA that plays a crucial role in the carcinogenesis of HCC by modulating the PTEN/PI3K/AKT and JAK-STAT3 signaling pathways [43,44]. MiR-221 also mediates EMT in HCC cells [45]. Sohn et al. detected higher serum levels of exosomal miR-221 in HCC patients than in liver cirrhosis patients [46]. We made similar observations in plasma. In our analyses, miR-221 was enriched in exosomes of HCC patients, but not in liver cirrhosis patients as compared with healthy individuals, illustrating the possible usefulness of miR-221 as a tumor marker for HCC screening in patients with liver cirrhosis.

We found that in patients with decompensated liver cirrhosis (with and without HCC), the levels of miR-146a, miR-192 and miR-221 were downregulated compared to patients with compensated liver cirrhosis. Waidmann et al. reported a similar effect of miR-122 which was downregulated in patients with decompensated liver cirrhosis and was an independent marker for OS [47]. However, in our study, a Cox regression model including MELD score and all three miRNAs showed that only MELD score was significantly associated with OS. Furthermore, as mentioned above, the exosomal levels of miR-16 were associated with impaired survival in patients with liver cirrhosis, but interestingly, miR-16 expression was not significantly different between patients with compensated or decompensated cirrhosis.

## 5. Conclusions

In summary, we identified three miRNAs that are significantly upregulated in HCC patients and could use these to discriminate between tumor patients, patients with liver cirrhosis and healthy individuals. Furthermore, we demonstrated the prognostic impact of miR-192 and miR146a in patients with early and intermediated stage HCC. In liver cirrhosis patients, we identified miR-16 as a prognostic marker that was associated with OS. Therefore, miR-16 may serve as a non-invasive surrogate parameter in future studies to predict progression of fibrosis in patients with chronic liver disease.

Collectively, our findings indicate the role of selected miRNAs as biomarkers for diagnosis, prognosis, and therapy monitoring in HCC patients. However, beyond that, the results of our study confirm that miRNAs are also prognostic markers in patients with liver cirrhosis and may, therefore, help to improve risk stratification in these patients.

## Figures and Tables

**Table 1:** Clinicopathological parameters of hepatocellular carcinoma (HCC) and liver cirrhosis (LC) patients (training cohort). The cohort included 24 HCC patients (median age: 65; range 44–79), 37 LC patients (59; 32–78) and 20 healthy controls.

Characteristics		HCC Patients	LC Patients
Gender (*n*; %)	Male	20 (83.3)	22 (59.5)
Etiology of LC	Alcoholic	9 (39.1)	20 (54.1)
	Non-alcoholic	14 (60.9)	17 (45.9)
MELD	≤20	24 (100)	25 (67.6)
	>20	0	12 (32.4)
CPS	A	9 (37.5)	6 (16.2)
	B	11 (45.8)	14 (37.8)
	C	4 (16.7)	17 (46.0)
**History of Decompensation**			
Ascites		9 (37.5)	27 (73)
HE		0	9 (24.3)
HRS		0	9 (24.3)
Variceal bleeding		0	3 (8.1)
**Tumor Characteristics**			
BCLC	A	5 (20.9)	n.a.
	B	10 (41.6)	n.a.
	C	9 (37.5)	n.a.
Distant metastases (*n*)		5 (20.8)	n.a.
Tumor nodules (*n*)		2 (1–5)	n.a.
Sum of largest diameter (cm)		7 (3–16)	n.a.
**Baseline Blood Results**			
WBC (10^9^/L)		6.2 (2.1–14.7)	7.4 (1.4–21.0)
Platelets (10^9^/L)		138 (33–298)	117 (11–354)
Albumin (g/L)		31 (19–39)	22 (10–34)
Bilirubin (mg/dL)		1.3 (0.2–6.1)	5.3 (0.5–30)
Creatinine (mg/dL))		1.0 (0.5–1.6)	1.5 (0.4–5.0)
GFR (ml/min)		81 (41–119)	65 (12–126)
AST (U/L)		67 (21–172)	91 (21–472)
ALT (U/L)		50 (18–108)	73 (13–767)
γGT (U/L)		290 (40–1378)	184 (27–1063)
CRP (mg/L)		22 (5–131)	32 (5–151)
AFP (kU/L)		2698 (2–23082)	8 (2–50)
INR		1.3 (1.0–1.9)	1.4 (1.0–2.3)
**Healthy Individuals (*n* = 20)**			
Gender (*n*, %)	Male: 12 (60)		

Abbreviations: AFP, alpha-fetoprotein; ALT, alanine aminotransferase; AST, aspartate aminotransferase; BCLC, Barcelona Clinic for Liver Cancer classification; CPS, Child Pugh Score; CRP, c-reactive protein; GFR, glomerula filtration rate; γGT, gamma-glutamyltransferase; HE, hepatic encephalopathy; HRS, hepato-renal syndrome; INR, international normalized ratio; MELD, model of end-stage liver disease; non-alcoholic etiology, including non-alcoholic fatty liver disease, hepatitis B, hepatitis C, autoimmune hepatitis, primary biliary cholangitis, primary sclerosic cholangitis; WBC, white blood cells.

**Table 2:** Clinicopathological parameters of HCC and liver cirrhosis (LC) patients analyzed using single TaqMan PCR (validation cohort). This cohort included 86 HCC patients (median age: 67; range 39–86), 51 LC patients (51; 21–78) and 20 healthy individuals (40; 20–67).

Characteristics		HCC Patients	LC Patients
Gender (*n*; %)	Male	74 (86)	33 (64.7)
Etiology of LC	Alcoholic	41 (47.7)	25 (49)
	Non-alcoholic:	45 (52.3)	26 (51)
	NAFLD	17 (19.8)	3 (5.9)
	HBV	7 (8.1)	3 (5.9)
	HCV	2 (2.3)	2 (3.9)
	PSC	0	2 (3.9)
	Others *	19 (22.1)	10 (19.6)
MELD	≤20	70 (96)	35 (71.4)
	>20	3 (4.1)	14 (28.6)
CPS	A	39 (45.3)	11 (21.6)
	B	29 (33.7)	20 (39.2)
	C	18 (20.9)	20 (39.2)
**History of Decompensation**			
Ascites		22 (25.9)	34 (66.7)
HE		2 (2.4)	13 (25.5)
HRS		1 (1.2)	9 (17.6)
Variceal bleeding		2 (2.4)	6 (11.8)
Total		23 (27.1)	38 (74.5)
**Tumor Characteristics**			
BCLC	A	7 (8)	n.a.
	B	39 (38)	n.a.
	C	40 (54)	n.a.
Distant metastases (*n*)		19 (22)	n.a.
Tumor nodules (*n*)		2 (1–6)	n.a.
Sum of largest diameter (cm)		7 (1–26)	n.a.
Progressive disease		38 (46.3)	n.a.
Overall survial	alive/dead	51/33	n.a.
**Baseline Blood Results**			
WBC (10^9^/L)		6.3 (1.4–14.7)	6.8 (1.1–21.0)
Platelets (10^9^/L)		155 (33–555)	120 (11-354)
Albumin (g/L)		30 (15–43)	24 (10–42)
Bilirubin (mg/dL)		1.5 (0.2–11.5)	4.7 (0.2–30)
Creatinine (mg/dL)		1.2 (0.5–9.0)	1.3 (0.4–5.0)
GFR (ml/min)		75 (6–119)	75 (12–151)
AST (U/L)		92 (11–2017)	83 (21–472)
ALT (U/L)		56 (16–810)	67 (13–767)
γGT (U/L)		262 (40–1421)	179 (14–1063)
CRP (mg/L)		20 (5–161)	27 (5–151)
AFP (kU/L)		11.961 (2–373.358)	8.4 (1.5–49.6)
INR		1.3 (0.9–11.0)	1.4 (1.0–2.3)

Abbreviations: AFP, alpha-fetoprotein; ALT, alanine aminotransferase; AST, aspartate aminotransferase; BCLC, Barcelona Clinic for Liver Cancer classification; CPS, Child Pugh Score; CRP, c-reactive protein; GFR, glomerula filtration rate; γGT, gamma-glutamyltransferase; HBV, hepatitis B virus related; HCV, hepatitis C virus related, HE, hepatic encephalopathy; HRS, hepato-renal syndrome; INR, international normalized ratio; MELD, model of end-stage liver disease; NAFLD, non-alcoholic fatty liver disease, PSC, primary sclerosic cholangitis..; WBC, white blood cells. * other non-alcoholic etiology, including autoimmune hepatitis, primary biliary cholangitis, secondary sclerosic cholangitis, hemochromatosis.

## Data Availability

The data presented in this study are available in this article (and supplementary material).

## Supplementary Materials

The following are available online at https://www.mdpi.com/article/10.3390/cancers13102484/s1, Figure S1: Hierachical cluster of 45 exosomal miRNA.; Figure S2: Significant deregulation of exosomal miRN-16, miR-146a, miR-192 and miR-221; Figure S3: Original Western Blot; Table S1: Training cohort: Significant changes in exosomal miRNA levels between HCC and LC patients, healthy individuals and distinct clinicopathological characteristics; Table S2: Training and validation cohort: Significant changes in exosomal miRNA levels between HCC and LC patients, healthy individuals assayed via miRNA array cards; Table S3: Pairwise spearman correlation comparing APF, miR-16, miR-146a, miR-192 and miR-221.
