# Supplementary material for: Diagnostic and Prognostic Value of miR-16, miR-146a, miR-192 and miR-221 in Exosomes of Hepatocellular Carcinoma and Liver Cirrhosis Patients
Source: Cancers (Basel). 2021 May 19;13(10):2484. doi: 10.3390/cancers13102484 (PMC8161187; doi:10.3390/cancers13102484)
Supplement: Supplementary file 1 [file cancers-13-02484-s001.zip › cancers-1184444-supplementary.pdf]

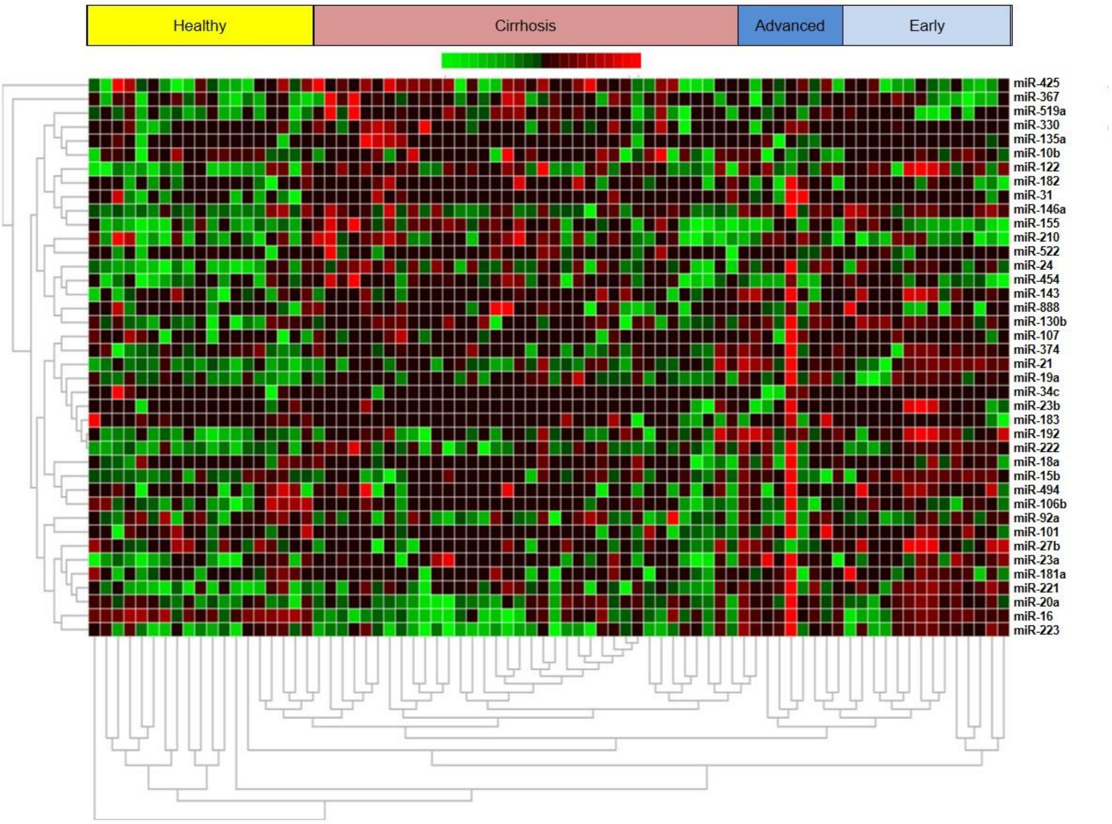

Figure S1. Hierarchical cluster of 45 exosomal miRNAs. A heat map of median centered ΔCq values of 45 exosomal miRNAs vs. the mean of references miR-484 and cel-miR-39 [in rows) derived from plasma samples of 24 HCC patients, 37 liver cirrhosis patients and 20 healthy individuals [in columns) is shown. Unsupervised hierarchical clustering of samples and miRNAs was based on average linkage and Pearson's correlation as a distance metric. The red and green colors indicate that the ΔCq values are below [relatively high expression) and above [relatively low expression levels) the median of all ΔCq values in the study, respectively. Bottom: clustering of samples. Left: clustering of probes. The scale bar provides information on the degree of regulation.

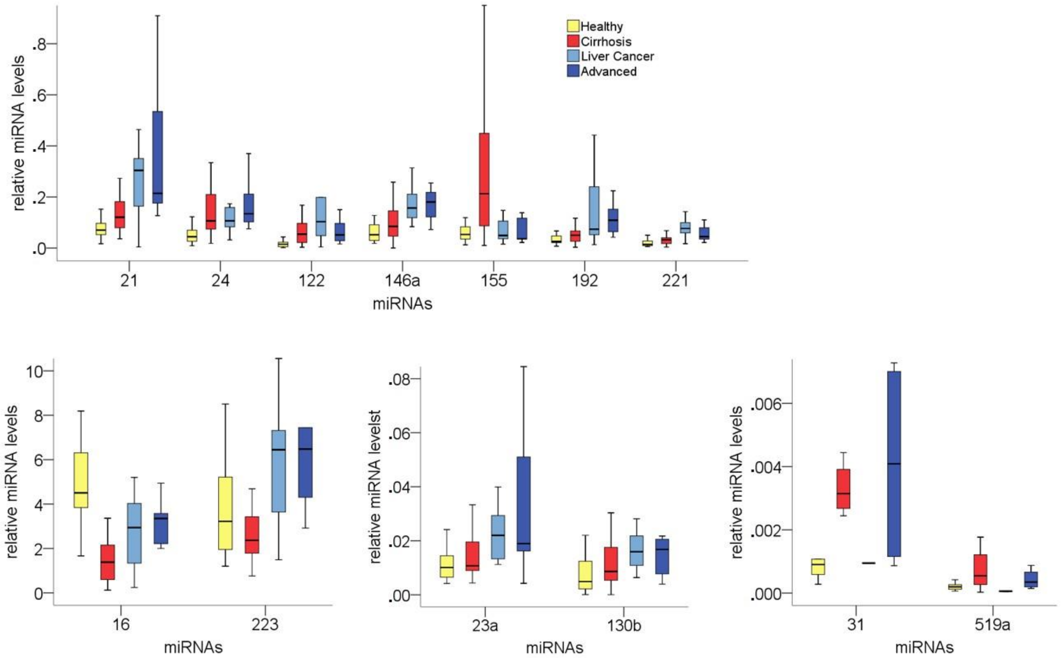

Figure S2. Significant deregulation of exosomal miR-16, miR-146a, miR-192 and miR-221. The box plots compare the significantly deregulated exosomal miRNA concentrations in the plasma of 24 HCC patients, 37 liver cirrhosis patients and

20 healthy individuals, as derived from the data of the array cards and volcano plots. The significant p-values of the statistical evaluations are summarized in Table S1 and Table S2.

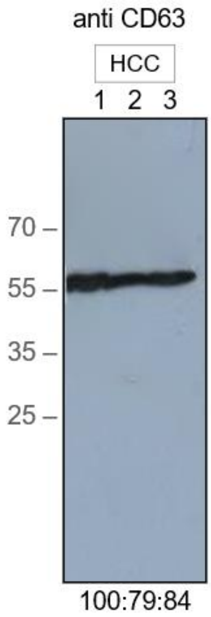

Figure S3. Original Western Blot: Exosomes were precipitated from 3 plasma samples of HCC patients by the agglutinating agent ExoQuick and analyzed by a Western blot using an antibody specific for the exosomal marker CD63. The percentages under the blot show intensities of the areas of three bands.

Table S1. Training cohort: Significant changes in exosomal miRNAs levels between HCC and LC patients, healthy individuals and distinct clinicopathological characteristics. Out of the 45 selected miRNAs that were tested via Array analysis, signifcant changes were only found in 13 miRNAs while the remaing 34 miRNAs revealed no relevant different expression. Abbreviations: , HE, hepatic encephalopathy; HRS, hepato-renal syndrome;; MELD, model of end-stage liver disease; VE, variceal haemorrhage.

miRNAs	HCC vs. Healthy	Cirrhosis vs. Healthy	HCC vs. Cirrhosis	MELD ≤20 vs. >20	CPS A vs. B (vs. C)	Ascites	HE	HRS	VE
miR-16		*0.0001*	*0.019*						
miR-21	*0.002*								
miR-23a	*0.018*			*0.013*	*0.078 (0.020)*		*0.007*		
miR-24	*0.002*	*0.005*					*0.004*		
miR-31		*0.026*							
miR-122	*0.005*	*0.026*							
miR-130b	*0.026*								
miR-146a	*0.002*		*0.026*		*0.024 (0.004)*		*0.031*		*0.008*
miR-155		*0.018*	*0.033*			*0.024*			
miR-192	*0.0001*		*0.033*					*0.017*	*0.034*
miR-221	*0.0004*		*0.012*						
miR-223			*0.007*						
miR-519a		*0.026*							

Table S2. Training and validation cohort: Significant changes in exosomal miRNAs levels between HCC and LC patients, healthy individuals assayed via miRNA array cards (training cohort; left column) and by using single TaqMan PCR (validation cohort; right column).

miRNAs	miRNA array cards			single TaqMan PCR		
	p-values (fold-changes)					
	HCC vs. Healthy	Cirrhosis vs. Healthy	HCC vs. Cirrhosis	HCC vs. Healthy	Cirrhosis vs. Healthy	HCC vs. Cirrhosis
miR-16		0.0001 (0.3)	0.019 (2.3)	0.0001	0.0001	0.015
miR-146a	0.002 (3.0)		0.026 (2.4)	0.0001		0.0001
miR-192	0.0001 (3.6)		0.033 (2.3)	0.002		0.078*
miR-221	0.0004 (3.8)		0.012 (2.4)	0.032		0.001
miR-21	0.002 (3.5)					
miR-23a	0.018 (2.2)					
miR-24	0.002 (3.0)	0.005 (2.5)				
miR-31		0.026 (3.6)				
miR-122	0.005 (5.0)	0.026 (3.2)				
miR-130b	0.026 (4.6)					
miR-155		0.018 (3.5)	0.033 (0.3)			
miR-223			0.007 (2.6)			
miR-519a		0.026 (2.9)				

Table S3. Pairwise spearman correlation comparing AFP, miRNA 16, miRNA 146a, miRNA 192 and miRNA 221.

	AFP	miRNA 16	miRNA 146a	miRNA 192	miRNA 221
**AFP**	1.00	- 0.1433	0.1957	0.0223	0.1324
**miRNA 16**	- 0.1433	1.00	0.0747	0.2728	0.2701
**miRNA 146a**	0.1957	0.0747	1.00	0.2434	0.4491
**miRNA 192**	0.0223	0.2728	0.2434	1.00	0.2248
**miRNA 221**	0.1324	0.2701	0.4491	0.2248	1.00
